# Surgical treatment of acute high‐grade acromioclavicular joint dislocations

**DOI:** 10.1002/jeo2.70173

**Published:** 2025-03-10

**Authors:** Theodorakys Marín Fermín, Chih‐Kai Hong, Lucca Lacheta, Lukas N. Münch, Knut Beitzel, Eoghan T. Hurley, Kai‐Lan Hsu, Emmanouil Brilakis, Berte Bøe, Davide Cucchi

**Affiliations:** ^1^ Clínica Santa Sofía Av. Principal de Santa Sofía El Cafetal Caracas Venezuela; ^2^ Thessaloniki Minimally Invasive Surgery (TheMIS) Orthopaedic Center St. Luke's Hospital Thessaloniki Panórama Greece; ^3^ Department of Orthopaedic Surgery, National Cheng Kung University Hospital, College of Medicine National Cheng Kung University Tainan Taiwan; ^4^ Department of Sports Orthopaedics, TUM University Hospital, Klinikum rechts der Isar Technical University of Munich Munich Germany; ^5^ ATOS Orthoparc Clinic Cologne Germany; ^6^ Sports Surgery Clinic Dublin Ireland; ^7^ Duke University Durham North Carolina USA; ^8^ 3rd Orthopaedic Department Hygeia Hospital Athens Greece; ^9^ Division of Orthopedics Surgery Oslo University Hospital Norway; ^10^ Department of Orthopaedics and Trauma Surgery Universitätsklinikum Bonn Bonn Germany

**Keywords:** acromioclavicular, coracoclavicular, dislocation, shoulder, sports injury

## Abstract

Treatment options for acute acromioclavicular joint (ACJ) instability include several surgical and non‐surgical approaches. Recent trends indicate a shift towards nonoperative treatment, even for severe Rockwood type V injuries, which traditionally required surgery. Despite this shift, some patients may still benefit from surgical stabilisation, particularly if significant pain and disability persist. Modern surgical techniques focus on cortical button systems and restoration of the coracoclavicular ligaments, emphasising the importance of the posterosuperior acromioclavicular capsuloligamentous complex in managing horizontal instability. Clavicular hook plates offer rigid stability but present risks, such as damage to the subacromial structures and acromial erosion. Although anatomical repair techniques have gained prominence due to their biomechanical advantages and have been endorsed by international societies, non‐anatomic methods may also provide acceptable outcomes with lower costs. The use of tendon grafts in chronic ACJ instability has shown promise, although evidence for their use in acute cases remains limited. This review discusses various treatment strategies, including operative and nonoperative management, focusing on patient outcomes, complication rates, and return‐to‐sport scenarios. Ultimately, the choice between surgical and non‐surgical treatment must consider individual patient needs and the potential for long‐term recovery.

**Level of Evidence**: Not applicable.

**Abbreviations**
AC: acromioclavicular
ACJ: acromioclavicular joint
ASES: American Shoulder and Elbow Surgeons
CC: coracoclavicular
CMS: Constant‐Murley score
DASH: disabilities of the arm, shoulder, and hand
ESA: European Shoulder Associates
ESSKA: European Society of Sports Traumatology, Knee Surgery and Arthroscopy
RTP: return to play
UCLA: University of California‐Los Angeles
VAS: visual analogue scale

## INTRODUCTION

Acromioclavicular joint (ACJ) dislocations are common in young adults and represent up to 50% of all shoulder sports injuries [5, 71]. The six‐types Rockwood classification [60] has been used to guide treatment, with high‐grade acute injuries (types III–VI)—< 3 weeks from trauma to surgery as proposed by the ISAKOS consensus statement [6]— being considered amenable for surgical treatment. Although these injuries have traditionally been managed operatively, recent studies suggest that nonoperative treatment can be successful in a high proportion of cases, with only approximately 15% of patients ultimately requiring surgery [24, 26, 31, 32, 46]. Based on the latest evidence, it appears therefore advisable to indicate surgery only for those patients who continue to experience restricted range of motion and unsatisfactory clinical scores 2–6 weeks post‐injury, a delay which does not appear to compromise surgical outcomes [2, 11, 31, 46, 53, 68].

In cases where surgery is indicated, various surgical techniques have been developed and over the years, ACJ instability treatment and research have focused on restoring the coracoclavicular (CC) ligament complex, utilising plates, screws or Kirschner wires, but modern cortical button system techniques later succeeded in this approach [51]. Recent studies have highlighted the importance of the posterosuperior acromioclavicular (AC) capsuloligamentous complex in managing the horizontal instability of the ACJ, and newer surgical techniques now emphasise the significance of AC fixation or ligament restoration [70]. Despite over 150 treatments for ACJ dislocations, no definitive evidence favours one technique over others [9]. This expert opinion review aims to discuss the state‐of‐the‐art management of acute high‐grade acromioclavicular joint dislocations surgical treatment.

## IS THERE A ROLE FOR HOOK PLATE FIXATION?

Clavicular hook plate internal fixation is a straightforward option for ensuring proper reduction, rigid fixation of ACJ dislocation, and effective healing, closely mimicking the original ACJ's stability [50, 77]. It promotes a short immobilisation period and allows early rehabilitation, leading to rapid healing of the conoid and trapezoid ligaments and quick recovery [20].

Despite its ease of application and reduced risk of intraoperative iatrogenic fractures associated with CC suspension devices [58], using a clavicular hook plate carries the risk of complications such as damage to the subacromial structures and, thus, is not considered a popular option in modern shoulder surgery anymore. The hook's placement narrows the subacromial space, often causing subacromial impingement [45]. Additionally, positioning the hook beneath the acromion can lead to acromial erosion [34], with incidence rates ranging between 62% and 100% [39, 56, 67]. Severe erosion may even result in acromial fractures [17]. Although symptoms typically resolve after implant removal, about 32% of patients experience a loss of reduction [43]. Nevertheless, clinical outcomes generally remain positive, indicating that the loss of reduction may not significantly affect functional results.

The morphology and the contact characteristics between the hook portion and the acromion are relevant factors affecting possible complications after hook plate fixation: [78] acromial and plate geometry, hook plate length [66], hook angle [35] and angle of implantation are relevant factors in the creation of stress risers on clavicle and acromion [65], advising for care when selecting the implant. On the other hand, plate thickness, screw number, and geometry are not expected to alter in a relevant way the implant's properties [42, 73].

**Figure 1:**
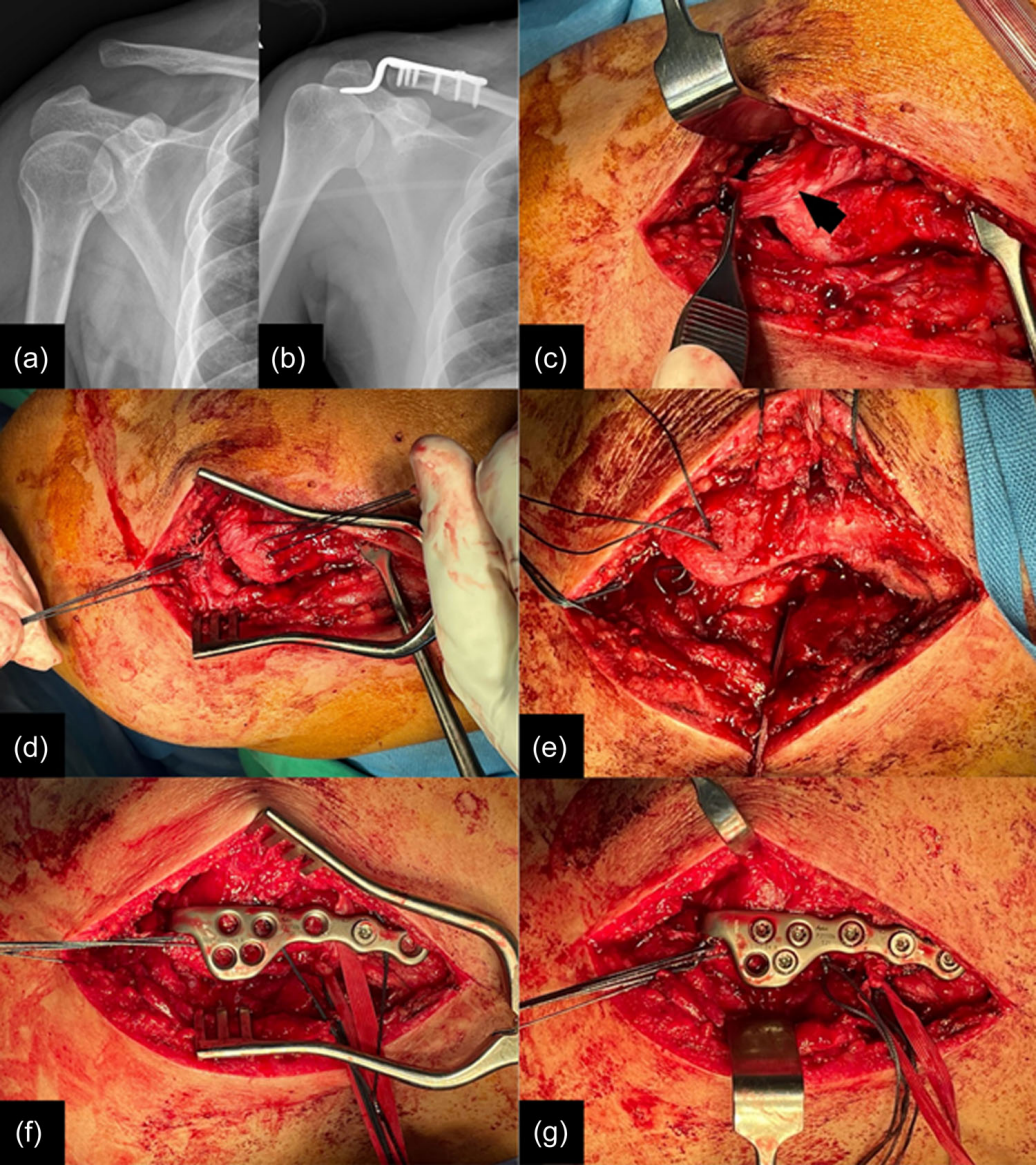
A 34‐year‐old man presented with a Type V acromioclavicular (AC) dislocation. The preoperative (a) and postoperative (b) plain films are shown. The surgical procedure was performed with the patient in the beach‐chair position. An incision was made directly over the AC joint. The AC ligament was found to be avulsed from the acromion (arrow) (c). The AC repair involved placing three transosseous sutures between the superior part of the acromion and the posterosuperior part of the distal clavicle (d). For coracoclavicular (CC) augmentation, one Mersilene tape and two No. 5 Ethibond sutures were used (e). The sutures for both the AC and CC repairs were initially left untied. A clavicular hook plate was then applied over the sutures, ensuring that the sutures were not visible in the screw holes (f). Once all the screws were secured, the sutures for the AC repair and CC augmentation were tied (g).

Additional CC ligament augmentation and possibly AC ligament repair are recommended to reduce the recurrence rate and lower the incidence of subacromial osteolysis (Figure 1a,b). Combining hook plate fixation with CC augmentation, this comprehensive approach has resulted in significantly less osteolysis than hook plate fixation alone [14, 16, 34, 40]. Consequently, patients experience lower pain levels, improved scores on the University of California‐Los Angeles (UCLA) shoulder scale, and better outcomes on the American Shoulder and Elbow Surgeons (ASES) score [14]. This technique demonstrates a marked superiority in terms of CC distance [16]. Such an approach should therefore be considered if hook plate fixation is chosen to treat acute ACJ instability, since the results of hook plate fixation, if pooled without specific selection of additional CC augmentation, have proved inferior to cortical button system techniques [3, 41, 44, 57, 74].

## NON‐ANATOMICAL VERSUS ANATOMICAL REPAIR

Non‐anatomic ACJ repair techniques aim to restore joint stability without considering the native anatomic ligament positioning or involving coracoacromial ligament transfer [7, 47]. These techniques were relegated during the last two decades, given that several biomechanical studies demonstrated that anatomical reproduction of CC and AC ligaments yielded superior stability [36, 49, 54, 64].

Cadaveric studies have demonstrated the relevance of the superior AC ligament complex on horizontal stability, as the CC ligaments do for vertical stability [22, 52], and while their specificity prompted replicating the native anatomy during these novel repair/reconstruction techniques, their clinical advantage has not proved superior [37, 69]. Recent evidence exposes comparable clinical outcomes, complications, failure, and return to sports rates between techniques, which may imply that recreating native ligament anatomy is not clinically relevant [27, 28, 38, 51, 75].

A systematic review by Moatshe et al. [51], including 34 studies (939 patients), revealed that all treatment modalities resulted in comparable subjective outcomes after surgical treatment of ACJ instability. Nevertheless, hook plates and K‐wires had the highest rate of complications (26.3%). Similarly, a systematic review by Xará‐Leite et al. [75], including 28 studies (799 patients) comparing non‐anatomic versus anatomic techniques, highlighted that both provided significant postoperative improvements with similar failure and reoperation rates.

Several implants and instrumentation were developed to meet the needs for anatomical techniques, leading to increased cost of implants over the years [25, 29, 47, 59]. Nevertheless, only a few studies have assessed the cost of ACJ repair, and those comparing the cost‐effectiveness highlight significant differences in consumables and materials between techniques [1, 33, 72].

A meta‐analysis by Marín Fermín et al. [47] comparing a total of 41 techniques revealed that although anatomic techniques yielded better biomechanical anterior stability and failure loads, non‐anatomic techniques or techniques combining two clavicular tunnels separated by at least 10 mm, a mean of two sutures, and/or suture tapes provided supraphysiological stability and failure loads at a lower cost of implants. However, the modified Weaver–Dunn technique—the former surgical standard—should be avoided because of suboptimal stability, failure load and high reoperation rates [8, 27, 47].

The current evidence has failed to prove that more stability translates into better patient‐reported outcomes or fewer complication rates. The comparable outcomes between anatomic and non‐anatomic techniques may be attributable to the supraphysiological stability they achieve in the laboratory setting. Furthermore, the equivalent loss of reduction between suture‐only fixation versus cortical button system compared with all other repair/reconstruction techniques favours suture‐based fixation [27]. Given their satisfactory outcomes and lower costs, non‐anatomic repair/reconstruction techniques can be considered a viable option in cost‐sensitive populations.

## IS THERE A ROLE IN USING A GRAFT IN THE ACUTE SETTING?

There is widespread agreement on using biological augmentation in the surgical treatment of chronic ACJ instability. After the reduction and fixation of an acute injury, the outcome depends on the healing of the patient's native ACJ capsule and the CC ligaments. A critical controversy is the time frame within which healing is expected to be feasible. The European Shoulder Associates (ESA) of the European Society of Sports Traumatology, Knee Surgery and Arthroscopy (ESSKA) 2020 consensus recommends a 2–3 week window for effective treatment [61], but other authors suggest this limit might extend to around 6 weeks [13, 18].

For Rockwood types III–V, the need for biological support for stabilisation increases as the time from injury lengthens, with the optimal window closing after 2–3 weeks (early acute phase). Some surgeons recommend surgical stabilisation without tendon grafts even if patients are seen between 3 and 6 weeks (delayed acute phase). While the ACJ capsule can often be repaired—with scar tissue interfering with reduction and capsule repair—CC ligament healing should not be expected in the latter and should be deemed unsuitable for repair.

There are limited studies on tendon reconstruction outcomes following acute ACJ dislocation. Lee et al. compared 12 acute cases treated with tendon grafts to 35 cases treated without grafts [40]. They found that repairs without tendon grafts resulted in 8 of 35 (23%) cases of loss of reduction, while those with tendon grafts resulted in 5 of 12 (42%) cases. This suggests a higher incidence of loss of reduction in the graft‐augmented group.

Treating ACJ instability in the delayed acute phase typically requires a tendon graft to reconstruct the CC ligaments. However, immediate postoperative reduction loss is frequent in follow‐ups. Whether this is due to graft stretching or insufficient horizontal stability remains unclear, but whenever possible, over‐reduction might be a favourable approach [48]. To date, no evidence or clinical experience supports using a biological graft in the acute setting of an ACJ dislocation.

## SURGICAL TECHNIQUES

### Hook plate fixation with CC augmentation

#### Surgical technique

The patient is positioned in the beach chair position. After sterile draping, a skin incision is made directly over the ACJ. The subcutaneous tissue is dissected to expose the joint. Given that the most biomechanically relevant part of the AC ligament originates from the superior part of the acromion and attaches to the posterosuperior part of the distal clavicle [55], transosseous sutures between the superior part of the acromion and the posterosuperior part of the distal clavicle for AC repair (Figure 1c,d).

Next, CC augmentation is performed using one Mersilene (Ethicon, USA) tape and two No. 5 Ethibond (Ethicon, USA) sutures. A shuttle suture is passed through the coracoid process using a curved wire passer, and all sutures are driven through with this shuttle suture (Figure 1e). The sutures for the AC and CC repairs are kept in place until the hook plate fixation is achieved.

The clavicular hook plate is then applied, with the hook placed under the acromion and the plate secured to the distal clavicle using a bone clamp. Care is taken to ensure that the sutures are not visible in the screw holes to avoid being damaged by the screws (Figure 1f,g). Once all the screws are secured, the sutures for the AC repair and CC augmentation are tied.

#### Postoperative care

The operated arm is immobilised in a sling for 2 weeks. Early shoulder range of motion exercises are encouraged during the postoperative period. Return to full load‐bearing capacity is allowed from the second month. The implants are removed at six months unless subchondral osteolysis is observed (keeping the AC and CC ligament augmentation sutures).

### Suture‐based acromioclavicular joint repair

#### Surgical technique

Surgery is performed open or arthroscopically assisted under general anaesthesia depending on concomitant injuries [23]. The patient is placed in the beach chair position and draped sterilely. Anatomic landmarks are identified and marked on the skin.

When a diagnostic arthroscopy is pursued, it is done in a standard fashion using a standard posterior viewing portal using a 70° arthroscope and an outside‐in anterolateral portal through the rotator interval. Subsequently, the arch and base of the coracoid process are carefully prepared with a radiofrequency device.

**Figure 2:**
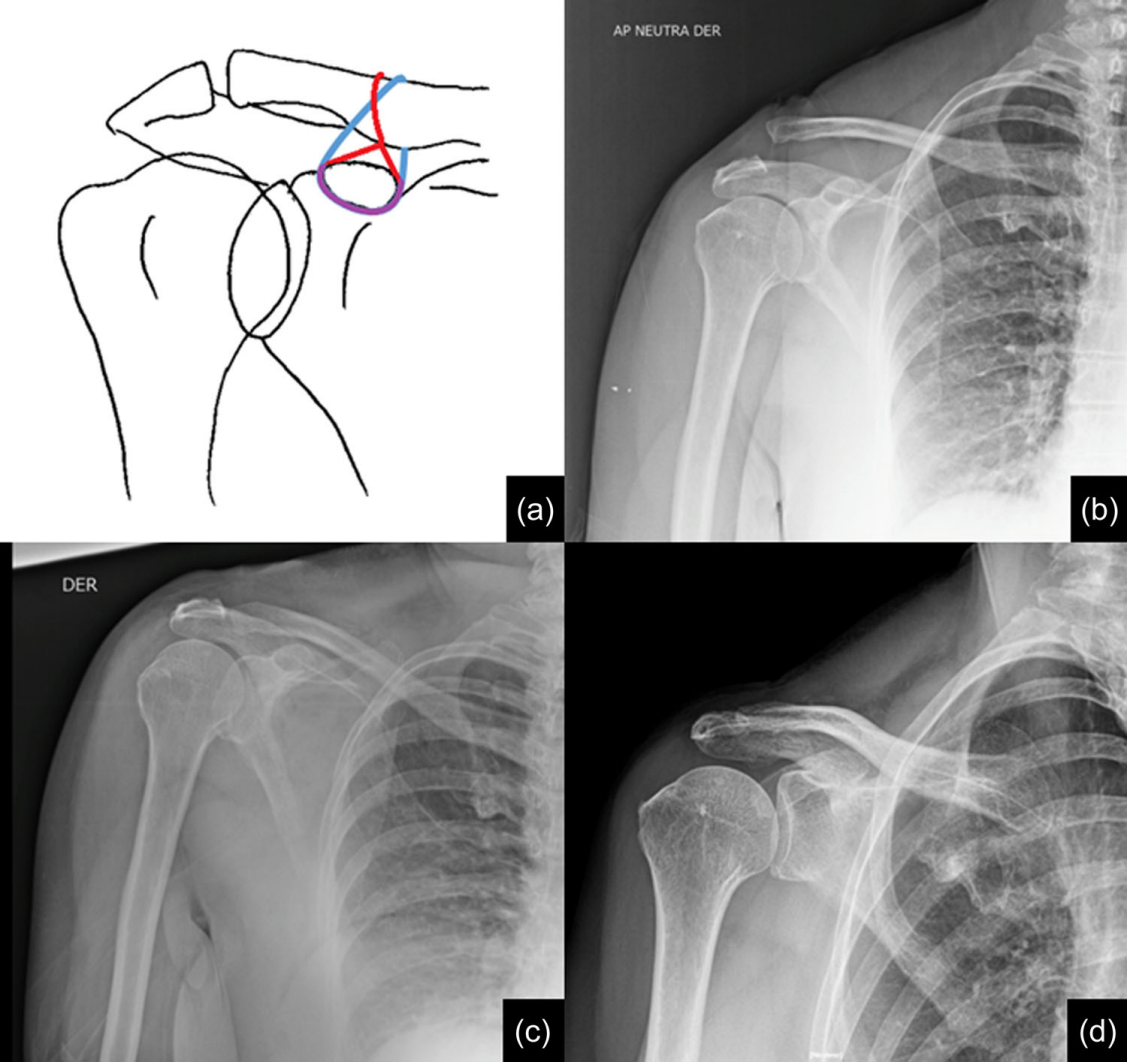
(a) Schematic representation of the suture‐based acromioclavicular joint repair. (b) Preoperative anteroposterior (AP) radiograph of a patient with an acute Rockwood V acromioclavicular joint (ACJ) right shoulder injury. (c) An immediate postoperative AP radiograph showed an overreduction of the ACJ. (d) 1‐year postoperative follow‐up AP radiograph.

Subsequently, a 3 cm transversal skin incision is made over the distal clavicle at the level of the coracoid process, around 3.5 cm from the ACJ, and dissected subperiostially, allowing an adequate identification of its anterior and posterior cortical margins. Anatomic reduction of the ACJ is managed and radiographically controlled using Kirschner wires or indirect manoeuvres, pushing the arm upward from the elbow. Via shuttle sutures (SutureLasso, Arthrex Inc., USA), two high‐strength suture tapes (FiberTape, Arthrex Inc., USA) are passed through clavicle incision under the coracoid (as close to its base as possible), one in a box‐configuration and the other in a figure‐eight‐configuration. Both are tightened using a Nice knot [30], starting with the box‐configuration one aiming for over‐reduction under intraoperative fluoroscopy. Incisions are then closed in a layered fashion, completing the surgical procedure (Figure 2a–d).

Suppose the procedure is to be performed in an open approach [21]. In that case, a 6 cm transversal skin incision is done over the distal clavicle at the level of the coracoid process in the same fashion, allowing the clavicle's anterior and posterior cortical margins identification. Next, CC augmentation is performed using two high‐strength suture tapes (FiberTape, Arthrex Inc., USA). A shuttle suture is passed through the coracoid process using a curved wire passer, and all sutures are driven through with this shuttle suture and tightened following the previously described configuration.

#### Postoperative care

The operated arm is immobilised in a sling for 3 weeks postoperatively. From there, daily living activities without load bearing are allowed, promoting active range of motion exercises at tolerance. Progressive load bearing is started with 2–5 kg for the following weeks. Full load bearing and return to play (RTP) are allowed 12 weeks after the procedure.

### Arthroscopically assisted anatomic acromioclavicular joint repair

#### Surgical technique

Surgery is performed arthroscopically assisted under general anaesthesia with an additional interscalene nerve block. The patient is placed in the beach chair position with the head slightly turned in the opposite direction to facilitate sufficient access to the clavicle. A thorough clinical examination is performed under anaesthesia to assess the severity of ACJ instability and evaluate the possibility of anatomic manual reduction of the ACJ. The operation field is prepared and draped in a sterile fashion; the arm is placed in a pneumatic arm holder. Anatomic landmarks are identified and marked on the skin.

First, a diagnostic arthroscopy is performed via a standard posterior viewing portal using a 30° arthroscope. The joint is carefully assessed for concomitant intraarticular injuries of the labrum, intraarticular course of the long head of the biceps tendon or rotator cuff, which are later addressed if necessary. Subsequently, an anterior working portal is established using an outside‐in technique through the rotator interval. The arch and base of the coracoid process are carefully prepared with an electrothermal ablation device and optimal visualisation of the coracoid base is ensured by switching the arthroscope to an additional lateral transtendinous viewing portal through the supraspinatus tendon [12]. The transtendinous portal should be placed directly posterior to the long head of the biceps tendon and lateral to the rotator cable.

Subsequently, a 3 cm skin incision is made over the ACJ in the orientation of the distal clavicle. The fascia is incised and carefully mobilised with elevation of the anterior and posterior flaps subperiosteally as a single layer to facilitate its later repair. Soft tissue impeding anatomic joint reduction (e.g., discus articularis) is then removed, followed by the peeling out of the AC ligaments. Consecutively, two horizontal tunnels are drilled through the distal clavicle and acromion, 10–15 mm, on either side of the ACJ using a 3.0 mm cannulated drill. Now, anatomic reduction of the ACJ is performed, radiographically controlled, and temporarily transfixed using a trans‐AC Kirschner wire. Via shuttle sutures (SutureLasso, Arthrex Inc., USA), a high‐strength suture tape (FiberTape, Arthrex Inc., USA) is passed through the drill holes in a box‐configuration [22].

**Figure 3:**
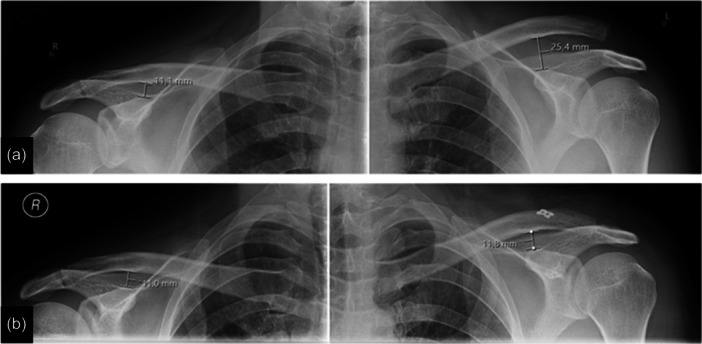
(a) Preoperative radiograph of a patient with an acute Rockwood V acromioclavicular joint (ACJ) left shoulder injury. (b) Arthroscopically assisted anatomic acromioclavicular and coracoclavicular stabilisation postoperative radiograph.

Then, a second small skin incision is placed over the clavicle at the anatomic position of the trapezoid and conoid ligament approximately 3.5 cm medial to the ACJ line. The trapezoid fascia is incised with exposure of the clavicle's anterior and posterior cortical margins. An ACJ drill guide (AC guide, Arthrex Inc., USA) is introduced via the anterolateral portal, and a transclavicular‐transcoracoid tunnel is established 3.5 cm medial to the ACJ line using a 3.0 mm cannulated drill. Given the oblique drilling direction, the entrance point of the transclavicular drill hole should be placed slightly posterior to the midline. Correct tunnel positioning at the base of the coracoid is verified under fluoroscopy. A shuttle wire replaces the drill. The free strands of two high‐strength suture tapes are shuttled retrograde through the coracoid and the clavicle via the anterior portal using the shuttle wire. The implant loops are attached to a titanium button (DogBone, Arthrex Inc., USA), and the button is positioned flush under the base of the coracoid process with the help of a grasper device. Correct button placement under the coracoid is visually controlled via the transtendinous portal. The four strands of the suture tapes are then threaded in a second titanium button on top of the clavicle. Next, the suture tapes are tightened and secured by alternating knots. Adequate reduction is then controlled via intraoperative fluoroscopy, while a slight over‐reduction is recommended. Finally, the ACJ‐cerclage is fixed by alternating knots, with the knot placed posterior to the ACJ for less soft tissue irritation. Incisions are then closed in a sterile fashion, completing the surgical procedure (Figure 3a,b) [12].

#### Postoperative care

The operated arm is immobilised in a sling for 6 weeks postoperatively. Only active‐assisted range of motion exercises are allowed during the first four weeks: the first and second weeks with abduction/adduction and flexion/extension 30°/0°/0° as well as internal/external rotation 80°/0°/15°; range of motion is then increased in the third and fourth week to abduction/adduction and flexion/extension 45°/0°/0° as well as internal/external rotation 80°/0°/30°. Later, in the fifth and sixth week, an active range of motion is allowed with abduction/adduction and flexion/extension 60°/0°/0°, as well as free internal/external rotation. At the seventh week postoperatively, a free active range of motion is allowed with a return to full load‐bearing capacity after 3 months.

## SUMMARY OF EVIDENCE

Alhtough surgical treatment has long been recommended for high‐grade ACJ instability, recent evidence supports an initial nonoperative approach, even for Rockwood type V injuries [24, 26, 31, 32, 46], which may benefit from nonoperative treatment without relevant functional drawbacks [15]. The current challenge lies in early identification of the subset of patients in whom conservative treatment is likely to fail, allowing timely surgical intervention [2, 11, 31, 46, 53, 68].

A potential treatment strategy involves early reassessment (two‐six weeks post‐injury): by this time, many patients will experience improved function and reduced pain. Those still experiencing significant pain and disability could then be considered for surgical stabilisation. This less aggressive approach is frequently dictated by careful clinical investigation and the differentiation of indications based on patients' pain, range of motion, and coping ability, variables that may not be evaluated in systematic reviews and meta‐analyses.

Bi et al. [10] conducted a network meta‐analysis involving 26 randomised controlled trials with 1581 patients suffering from acute Rockwood types III–V ACJ dislocations. They categorised the treatments into ten distinct groups: nonoperative treatment, Kirschner wire fixation, CC screw fixation, hook plate, open CC cortical button system, arthroscopic CC cortical button system, double CC cortical button system, isolated graft reconstruction, cortical button system with graft augmentation, and CC and ACJ fixation. Their findings indicated that AC fixation, cortical button system with graft augmentation, isolated graft reconstruction, double CC cortical button system, arthroscopic CC cortical button system, and open CC cortical button system were superior to hook plate, CC screw fixation, Kirschner wire fixation, and nonoperative treatment based on the Constant–Murley score (CMS) and Disabilities of the Arm, Shoulder, and Hand (DASH) score at the final follow‐up. Isolated graft reconstruction provided the best postoperative pain relief, measured by the visual analogue scale (VAS) score. Additionally, hook plate, double CC cortical button system, cortical button system with graft augmentation, AC fixation, arthroscopic CC cortical button system, and open CC cortical button system showed superiority in the CC distance and recurrence rates at final follow‐up, while Kirschner wire fixation and CC screw fixation had the shortest operative times, and isolated graft reconstruction and arthroscopic CC cortical button system the longest.

The results demonstrate that among the various treatment options for Rockwood types III‐V acute ACJ dislocations, CC fixation using cortical button system fixation, along with graft augmentation or additional AC fixation and/or ligament reconstruction, offers improved pain relief, functional outcomes, and reduced recurrence rates. AC fixation and graft augmentation of CC fixation provided the best outcomes in terms of CMS, DASH score, recurrence rate, and final CC distance. This success is likely due to the initial stability provided by CC fixation and the subsequent biological healing of the CC and AC ligaments and capsule when grafts or additional AC‐focused treatments are used.

The study underscores that while the CC ligaments are crucial for superior‐inferior restraint, the AC ligaments are essential for horizontal stability. Not addressing the AC ligaments when treating acute ACJ dislocations—especially in Rockwood IIIB injuries—can potentially lead to poorer outcomes [6, 61]. Moreover, older treatment methods were found to be inferior to newer surgical options. This might be related to the clavicle's physiological relative motion and rotation during scapular movement, which allows for better kinematics than in more rigid constructs. While the initial mechanical fixation with hook plate maintained a final CC distance comparable to CC suspensory fixation with or without graft or AC fixation, the screw and Kirschner wire fixation techniques were less effective, performing poorly in VAS, CMS, and DASH scores compared to other surgical treatments.

Although no specific meta‐analysis comparing hook plate fixation with CC augmentation and cortical button system techniques exists, if hook plate fixation is chosen to treat acute ACJ instability, additional CC augmentation is therefore recommended since, in general, the results of hook plate fixation have proved inferior as compared to alternative, cortical button system techniques [3, 41, 44, 57, 74].

Hook plate fixation and cortical button fixation system techniques have shown comparable efficacy in restoring shoulder function and relieving pain for acute Rockwood types III–VI dislocations. However, in recent meta‐analyses, cortical button fixation system techniques have yielded slightly better CMS and lower VAS scores than hook plate fixation, suggesting improved functional recovery and comfort. Two studies by Arirachakaran et al. [3, 4] highlighted that cortical button fixation system techniques not only result in higher CMS but also present a lower VAS, reinforcing the efficacy of suspensory devices in acute, high‐grade injuries. Nevertheless, cortical button fixation system techniques showed a higher overall complication rate (1.69 times greater than hook plate), possibly due to device positioning and tensioning risks. This increased complication risk emphasises the need for precise surgical techniques and may limit the applicability of cortical button fixation system in settings where simplicity of fixation is preferred.

A further meta‐analysis of four studies by Pan et al. [57], including 179 patients, indicated that, while both techniques effectively manage pain, the cortical button fixation system was associated with significantly lower VAS pain scores postoperatively, although CMS differences were not statistically significant. Cortical button fixation system allows for a more physiological ACJ movement, which could explain the pain reduction observed. This tendency was confirmed in a recent systematic review by Li et al. [44], comparing cortical button system and hook plate fixation techniques for acute ACJ dislocation in fourteen studies with a total of 795 patients (363 treated with SB and 432 with hook plate). Five of the 13 studies reported significantly higher CMS for the cortical button system group, with most of these studies utilising an arthroscopic cortical button system technique, which may enhance shoulder mobility and reduce joint stiffness. Regarding pain relief, three of the seven studies reported lower VAS scores in the cortical button system group, although the differences did not reach the minimal clinically important difference, suggesting that while cortical button system may offer better pain control, the effect size is limited.

Regarding recurrent instability, no statistically significant differences were observed between the cortical button system and hook plate groups, indicating comparable stability postoperatively [44]. A notable advantage of the cortical button system technique was lower blood loss, as reported across all studies, likely due to its less invasive nature than hook plate fixation. No significant differences were found in terms of other surgical metrics, such as operation time, postoperative CC distance, and overall complication rates [44].

Similar findings were published by Xie et al. [76] in a systematic review of 16 studies (471 patients in the cortical button system group and 445 patients in the hook plate group), concluding that cortical button system may lead to higher CMS and lower VAS scores. In terms of postoperative management, A further aspect to be considered when choosing the approach is that intraarticular‐associated lesions after acute ACJ injuries have a prevalence of around 20%, and up to a quarter of them require further surgical treatment which can be performed simultaneously with arthroscopy [62].

Hook plate fixation generally requires a second surgery for implant removal due to hardware discomfort or interference with joint movement, a step that is typically unnecessary with cortical button system, although cases of cortical button system removal with subsequent loss of reduction were described [63]. For high‐grade acute injuries with surgical indication, consensus guidelines from ESA‐ESSKA recommend minimally invasive anatomic reconstruction with a suspensory device for joint stability in the short term, without the need for biological augmentation in most cases [61].

### Return to play

RTP at the pre‐injury level is a critical expectation for athletes undergoing shoulder surgery. Cleary et al. [19] identified 120 studies involving 4327 athletes with Grade III–V ACJ dislocations. The most common sport associated with these injuries was cycling. The overall RTP rate was 91.5%, with 85.6% returning to the same level of play after nonoperative treatment, Kirschner wire fixation, CC screw fixation, hook plate, open CC cortical button system, arthroscopic CC cortical button system, double CC cortical button system, isolated graft reconstruction, cortical button system with graft augmentation, and CC or ACJ fixation. Similarly, for collision athletes, the RTP rate was 97.3%, with 97.2% returning to the same level.

In contrast, overhead athletes had a 97.1% RTP rate, but only 79.2% returned to the same level. The average time to RTP was 5.7 months, ranging from 1.5 to 15 months. Type III injuries had the highest RTP rate at 98.7% and the earliest RTP at 4.9 months, while types IV and V had RTP rates above 90%. Surgical techniques such as Kirschner wire fixation, isolated graft reconstruction, cortical button system with graft augmentation, AC fixation, cortical button system, and hook plate all reported RTP rates above 90%, with isolated graft reconstruction allowing the earliest RTP at 3.6 months, followed by AC fixation at 4.4 months.

The key finding from the Cleary et al. [19] review was that surgical treatment for ACJ dislocation generally leads to a high RTP rate, irrespective of the specific treatment used. However, significant differences were noted between overhead and collision athletes in terms of returning to the preinjury level. Almost all collision athletes returned to their prior level of play, whereas over 20% of overhead athletes did not. The reasons for not returning to the same level of play were not specified but are likely to be varied and subjective, including factors beyond physical limitations, such as lifestyle changes, decisions to retire from professional sports, and fear of reinjury.

## CONCLUSION

The management of acute high‐grade acromioclavicular joint dislocations is continually evolving, with both surgical and non‐surgical options showing potential depending on patient‐specific factors. Current evidence supports an initial nonoperative approach for high‐grade acromioclavicular dislocations, with close follow‐up to identify patients with persistent motion restrictions and unsatisfactory clinical results, allowing timely surgical intervention in these cases. While modern surgical techniques such as the cortical button system and acromioclavicular ligament repair have biomechanical advantages, non‐anatomical methods may also yield satisfactory results at a lower cost. Ultimately, treatment strategies should ideally be individualised based on injury severity, patient activity levels, and long‐term recovery goals.

## AUTHOR CONTRIBUTIONS

**Theodorakys Marín Fermín**: Conceptualization, methodology, validation, formal analysis, investigation, resources, data curation, writing–original draft, writing–review and editing, visualization, project administration. **Chih‐Kai Hong**: Conceptualization, methodology, validation, formal analysis, investigation, resources, writing–original draft, writing–review and editing, visualization. **Lucca Lacheta**: Writing–review and editing, supervision. **Lukas N. Münch**: Writing–review and editing, supervision. **Knut Beitzel**: Investigation,writing–review and editing. **Eoghan T. Hurley**: Investigation, writing–review and editing. **Kai‐Lan Hsu**: Writing–review and editing, supervision. **Emmanouil Brilakis**: Writing–review and editing, supervision. **Berte Bøe**: Writing–review and editing,supervision. **Davide Cucchi**: Conceptualization,methodology, validation,formal analysis, investigation, resources,data curation, writing–original draft, writing–review and editing, visualization, supervision, project administration, funding acquisition.

## ETHICS STATEMENT

No ethical approval was required for the presented study. No informed consent was required for the presented study.

## CONFLICT OF INTEREST STATEMENT

The authors declare no conflicts of interest relevant to the content of this review.

## Data Availability

The data underlying this article are available in the article and its online supplementary material.
